# Tumour marker antigen CA125 in pancreatic cancer: a comparison with CA19-9 and CEA.

**DOI:** 10.1038/bjc.1986.259

**Published:** 1986-12

**Authors:** C. Haglund

## Abstract

CA125 is a tumour marker test based on a monoclonal antibody against an antigen from an ovarian carcinoma cell line. Serum concentrations of CA125 were determined in 95 patients with pancreatic cancer and in 106 patients with benign pancreatic, biliary and hepatocellular diseases. The CA125 concentrations were compared with the CA19-9 and CEA levels. Almost half (45%) of the patients with pancreatic cancer had an elevated CA125 level (greater than 35 U ml-1). Elevated values were also found in benign diseases (24%), especially in patients with pancreatitis and benign hepatocellular diseases, but more seldom in extrahepatic cholestasis. It seems that CA125 is of limited value in the diagnosis of pancreatic cancer. Combination of the CA125 with the CA19-9 test increases the sensitivity only 6% as compared to the CA19-9 assay alone. There may, however, be a use for CA125 in differentiating between obstructive jaundice of benign and malignant origin.


Br. J. Cancer (1986), **54**, 897–901

### Tumour marker antigen CA125 in pancreatic cancer: A comparison with CA19-9 and CEA

C. Haglund

*Fourth Department of Surgery, Helsinki University Central Hospital, Kasarmikatu 11-13, SF-00130 Helsinki, Finland.*

**Summary** CA125 is a tumour marker test based on a monoclonal antibody against an antigen from an ovarian carcinoma cell line. Serum concentrations of CA125 were determined in 95 patients with pancreatic cancer and in 106 patients with benign pancreatic, biliary and hepatocellular diseases. The CA125 concentrations were compared with the CA19-9 and CEA levels. Almost half (45%) of the patients with pancreatic cancer had an elevated CA125 level ($>35\,U\,ml^{-1}$). Elevated values were also found in benign diseases (24%), especially in patients with pancreatitis and benign hepatocellular diseases, but more seldom in extrahepatic cholestasis. It seems that CA125 is of limited value in the diagnosis of pancreatic cancer. Combination of the CA125 with the CA19-9 test increases the sensitivity only 6% as compared to the CA19-9 assay alone. There may, however, be a use for CA125 in differentiating between obstructive jaundice of benign and malignant origin.

The CA125 assay is a new cancer test based on a monoclonal antibody OC125, which was originally raised against an epithelial ovarian cancer cell line (Bast *et al.*, 1981, 1983). The structure of the antigenic determinant is not completely defined, but in serum it appears to be associated with a high molecular weight mucin-like glycoprotein. An immunoradiometric assay has been developed to measure the concentrations of CA125 (Bast *et al.*, 1983, Klug *et al.*, 1984). High concentrations of CA125 have been found in more than 80 percent of sera from patients with ovarian cancer, and the test is especially promising in the follow-up of these patients (Bast *et al.*, 1983, 1984; Canney *et al.*, 1984). Elevated serum CA125 levels have also been found in patients with various gastrointestinal cancers, especially in patients with pancreatic cancer (59%) (Bast *et al.*, 1983; Klug *et al.*, 1984). Bast *et al.* (1983) recorded elevated serum levels ($>35\,U\,ml^{-1}$) in only one percent of healthy individuals and six percent of patients with non-malignant diseases. Ruibal *et al.* (1984), however, have reported considerably elevated CA125 values in patients with various benign diseases, such as liver cirrhosis (64%), liver granulomatosis (44%), pancreatitis (38%) and peritonitis (75%).

In this study, the utility of the CA125 test in the diagnosis and monitoring of patients with pancreatic cancer was evaluated. The serum levels from patients with pancreatic cancer were compared with those from patients with benign pancreatic, biliary tract and hepatocellular diseases. The CA125 levels were also compared with the concentrations of CA19-9 and CEA.

Received 9 June 1986; and in revised form 11 August 1986.

### Patients and methods

### *Patients*

Preoperative serum samples were obtained from 95 patients with pancreatic cancer (9 resectable, and 86 locally spread or metastasized tumours), including two islet cell carcinomas, one carcinoid tumour, three cystadenocarcinomas, 2 anaplastic carcinomas, 14 poorly differentiated and 37 well-to-moderately differentiated ductal adenocarcinomas. The exact degree of differentiation could not be determined from available cytological samples in 36 patients with an adenocarcinoma. Serial samples were obtained from three radically operated patients, who later developed a recurrence, and from 7 patients with a nonresectable tumour. Patients who had received chemotherapy or radiotherapy to the pancreatic region were not included in the study.

Fifty-two patients had a benign pancreatic disease: severe haemorrhagic pancreatitis (25 patients), non-haemorrhagic acute pancreatitis (17), acute pancreatitis associated with pseudocyst formation (2), and chronic pancreatitis (8). Thirty-one patients had benign biliary tract diseases: cholestasis due to stones in the common bile duct (13 patients) or postoperative stenosis (1), stones in the bile ducts without jaundice (4), and gallbladder stones (13). Hepatocellular jaundice was due to hepatic cirrhosis (9 patients), acute alcoholic hepatitis (2), or viral hepatitis (12).

© The Macmillan Press Ltd., 1986

*Assays*

The serum samples were stored at −20°C or −70°C from one to 30 months until assayed. The CA125 concentration in serum was measured using the Abbott CA125 RIA Diagnostic kit (Abbott, Wiesbahn, West Germany), and the recommended cut-off level of 35 U ml$^{-1}$ was used (Bast *et al.*, 1983; Klug *et al.*, 1984). The CA19-9 concentration was measured using the CA19-9 RIA kit (Centocor, Malvern, PA, USA), and the CEA concentration by a double antibody radioimmunoassay (Rutanen *et al.*, 1978), or using the Abbott-CEA RIA Diagnostic kit (Abbott, Wiesbahn, West Germany). The cut-off values of 37 U ml$^{-1}$ and 2.5 ng ml$^{-1}$ were used for CA19-9 and CEA, respectively. The results of the CA19-9 and CEA assays in pancreatic cancer and in benign pancreatico-biliary diseases have been described earlier (Jalanko *et al.*, 1984, Haglund *et al.*, 1986).

**Figure 1** Serum CA 125 concentrations in patients with localized (□) and advanced (●) pancreatic cancer; and with acute (○) and chronic pancreatitis (△); benign biliary diseases with (○) and without (□) jaundice and hepatocellular diseases. The cut-off value 35 U/ml for the CA 125 test is indicated by a dashed line.

### Results

*CA125 in pancreatic cancer*

Forty-three of the 95 patients with pancreatic cancer (45%) had a serum CA125 concentration above 37 U ml$^{-1}$, and 31% higher than 65 U ml$^{-1}$ (median 28 U ml$^{-1}$, range 7.6–5700 U ml$^{-1}$)(Figure 1, Table I).

High levels were found especially in patients with widely disseminated cancers. Only 2 out of 9 patients with a resectable pancreatic tumour had slightly elevated values (Figure 1). The concentration was increased in 14 patients (38%) with a well-to-moderately differentiated adenocarcinoma, in 8 patients (50%) with a poorly differentiated or an anaplastic carcinoma, and in 1 of 3 patients with a cystadenocarcinoma. The 2 patients with an islet cell carcinoma and the patient with a carcinoid tumour had normal CA125 levels.

The serum CA125 levels were serially determined in 9 cancer patients. Three patients, who underwent radical surgery had a normal CA125 level before operation. In two of these patients the serum level increased after clinical detection of recurrence (Figure 2). The CA125 concentration increased with tumour progression in 2 patients after palliative by-pass operation, whereas 3 patients had a normal serum level before and after surgical treatment. The level decreased during the follow-up in one patient treated conservatively.

*CA125 in benign diseases*

Thirteen of the 52 patients (25%) with pancreatitis

**Table I** Serum CA125 concentrations in patients with pancreatic cancer and with benign pancreatic, biliary tract and hepatocellular diseases

| | | *CA125* | | |
|---|---|---|---|---|
| *Diagnosis* | *No. tested* | *>35 U ml$^{-1}$* [a] | *>65 U ml$^{-1}$* [b] | *>350 U ml$^{-1}$* |
| Pancreatic cancer | 95 | 45% | 31% | 16% |
| Pancreatitis | 52 | 25% | 17% | 0% |
| Benign biliary disease | 31 | 13% | 7% | 0% |
| Hepatocellular jaundice | 23 | 35% | 22% | 9% |

[a]99% confidence limit for healthy blood donors; [b]99.8% confidence limit for healthy blood donors.

**Figure 2** Serial CA125 levels in 3 patients, who underwent pancreatico-duodenectomy for pancreatic cancer, and developed a recurrence. The arrows indicate the time of clinical verification of the recurrence.

had a slightly increased CA125 concentration (median $13.5\,U\,ml^{-1}$, range: $<7.6$–$311\,U\,ml^{-1}$). Only one of 8 patients with chronic pancreatitis showed a slightly elevated CA125 level. (Figure 1, Table I).

Elevated CA125 levels were found in 4 patients (13%) with benign biliary disease (median $9.0\,U\,ml^{-1}$, range: $<7.6$–$315\,U\,ml^{-1}$). Two patients had a stone in the common bile duct, one with and the other without jaundice, and 2 had acute cholecystitis with jaundice. (Figure 1, Table I).

Seven patients with liver cirrhosis and one patient with viral hepatitis had an increased CA125 concentration (35%, median $19\,U\,ml^{-1}$, range: $<7.6$–$680\,U\,ml^{-1}$)(Figure 1, Table I).

No correlation between the CA125 concentration and the bilirubin ($r=-0.06$), alkaline phosphatase ($r=0.03$), amylase ($r=-0.03$) or GOT ($r=0.06$) levels were seen in this material.

### *Comparison of CA125, CA19-9 and CEA*

The CA125 levels did not correlate with those of CA19-9 ($r=0.06$)(Figure 3) or CEA ($r=0.23$). The assay parameters for the CA125, CA19-9 and CEA assays, and for the combinations of the tests are summarized in Table II.

**Table II** Assay parameters for the CA125, CA19-9 and CEA assays, and for the combinations of the tests

| *Assay parameters* | *CA125+*[a] | *CA19-9+*[b] | *CEA+*[c] | *CA125 or CA19-9+* | *CA125 or CEA+* | *CA125 and CA19-9+* | *CA125 and CEA+* | *CA125, CA19-9 and CEA+* |
|---|---|---|---|---|---|---|---|---|
| Sensitivity[d] | 45% | 78% | 54% | 84% | 64% | 41% | 36% | 33% |
| Specificity[e] | 76% | 78% | 76% | 60% | 56% | 93% | 93% | 99% |
| Predictive value[f] | | | | | | | | |
| positive | 63% | 75% | 68% | ND | ND | 85% | 84% | 97% |
| negative | 61% | 81% | 81% | ND | ND | 64% | 59% | 59% |

[a]CA125+: $>35\,U\,ml^{-1}$; [b]CA19-9+: $>37\,U\,ml^{-1}$; [c]CEA+: $>2.5\,ng\,ml^{-1}$; [d]Sensitivity=TP/(TP+FN); [e]Specificity=TN/(TN+FP); [f]Predictive value, positive=TP/(TP+FP), negative=TN/(TN+FN). TP=true positive; FN=false negative; TN=true negative; FP=false positive; ND=not determined.

**Figure 3** Comparison of the CA125 and CA19-9 concentrations in patients with pancreatic cancer (●) and with benign pancreatic, biliary tract and hepatocellular diseases (○). The cut-off values for the assays are marked as dashed lines.

### Discussion

Elevated CA125 levels are mainly found in serum of patients with epithelial ovarian cancer (82%), but are also reported in 59% in pancreatic cancer and in 12–32% in various other non-gynaecological cancers (Bast *et al.*, 1981, 1983; Klug *et al.*, 1984). In this material less than half (45%) of the patients with pancreatic cancer showed a value over 35 U ml$^{-1}$. By using a higher cut-off level of 65 U ml$^{-1}$ (0.2% of healthy blood donors, Bast *et al.*, 1983) to increase the specificity, the sensitivity fell to as low as 31%. Only 8% of the patients with pancreatic cancer had a serum CA 125 concentration higher than any patient with benign disease. Thus, the low sensitivity limits the use of the CA125 test in the diagnosis of pancreatic cancer.

The control group represented differential diagnostic problems in clinical practice. Elevated values were found in 24% of these patients, especially in those with benign liver diseases, as well as in some patients with pancreatitis. Interestingly, benign extrahepatic cholestasis, which is the main cause of false positive values of the CA19-9 and CEA tests, was only seldom associated with elevated CA125 levels.

No correlation between the CA125, CA19-9 and CEA tests was found. Using the recommended cut-off values the CA125, CA19-9 and CEA tests showed a similar specificity. The sensitivity of the CA19-9 assay was clearly highest, but a combination of the CA125 test with the CA19-9 assay increased the sensitivity only very little, while the specificity clearly decreased. A specificity close to 100% (99%) is achieved if an increased concentration of all three markers occurs. Unfortunately, only one third of the cancer patients had an elevated level of all markers, which is too little for use in clinical practice.

The source of CA125 in serum of patients with pancreatic cancer is not known. Immunohistochemical staining technique is an appropriate method of studying the tissue expression of various cancer-associated antigens (Haglund *et al.*, 1986). However, the CA125 antigen seems to be destroyed during paraffin embedding of the tissue specimens (Kawabat *et al.*, 1983, Haglund *et al.*, unpublished). Four cryosections from pancreatic carcinomas were stained, out of which one specimen expressed CA125 (Haglund *et al.*, unpublished).

Clearly elevated serum levels of CA125 were seen only in patients with disseminated disease. This may be due to a large tumour burden, or may reflect liver involvement, as many of the patients with benign hepatocellular diseases showed elevated CA125 levels. This is in concordance with the findings of Ruibal *et al.* (1984), who reported elevated values in patients with liver cirrhosis, liver granulomatosis, hepatomas and metastatic liver disease. The elevated serum CA125 levels in liver processes may be due to a production of CA125 in the liver itself or may be caused by a defect of the liver to metabolize the antigen. It is also possible that CA125 may partly originate from the peritoneum in patients with ascites due to benign liver disease or metastatic disease. The peritoneum, especially areas of inflammation, is known to express CA125 (Kawabat *et al.*, 1983) and 75% of patients with peritonitis have elevated serum CA125 levels (Ruibal *et al.*, 1984).

It seems that CA125 is of little value as a tumour marker in the primary diagnosis and follow-up of patients with pancreatic cancer. A combination of CA125 with CA19-9 does not increase the sensititivity of the CA19-9 assay. However, in patients with obstructive jaundice the CA125 assay may be helpful in differentiating between malignant and benign processes.

This study has been supported by grants from Finska Läkaresällskapet, The Finnish Cancer Society, Svenska Kulturfonden, and the Oskar Öflund Foundation.

**References**

BAST, R.C. Jr., FEENEY, M., LAZARUS, H., NADLER, L.M., COLVIN, R.B. & KNAPP, R.C. (1981). Reactivity of a monoclonal antibody with human ovarian carcinoma. *J. Clin. Invest.* **68,** 1331.

BAST, R.C. Jr., KLUG, T.L., ST JOHN, E. & 9 others. (1983). A radioimmunoassay using a monoclonal antibody to monitor the course of epithelial ovarian cancer. *N. Engl. J. Med.,* **309,** 883.

BAST, R.C. Jr., KLUG, T.L., SCHAETZL, E. & 5 others. (1984). Monitoring human ovarian carcinoma with a combination of CA125, CA19-9, and carcinoembryonic antigen. *Am. J. Obstet. Gynecol.* **149,** 553.

CANNEY, P.A., MOORE, M., WILKINSON, P.M. & JAMES, R.D. (1984). Ovarian cancer antigen CA125. A prospective clinical assessment of its role as a tumour marker. *Br. J. Cancer,* **50,** 765.

HAGLUND, C., LINDGREN, J., ROBERTS, P.J. & NORDLING, S. (1986). Gastrointestinal cancer associated antigen CA19-9 in histological specimens of pancreatic tumours and pancreatitis. *Br. J. Cancer,* **53,** 189.

HAGLUND, C., ROBERTS, P.J., KUUSELA, P., SCHEININ, T.M., MÄKELÄ, O. & JALANKO, H. (1986). Evaluation of CA19-9 as a serum tumour marker in pancreatic cancer. *Br. J. Cancer,* **53,** 197.

JALANKO, H., KUUSELA, P., ROBERTS, P., SIPPONEN, P., HAGLUND, C. & MÄKELÄ, O. (1984). Comparison of a new tumour marker CA 19-9™, with alpha-fetoprotein and carcinoembryonic antigen in patients with upper gastrointestinal diseases. *J. Clin. Path.,* **37,** 218.

KAWABAT, S.E., BAST, R.C. Jr., BHAN, A.K., WELCH, W.R., KNAPP, R.C. & COLVIN, R.B. (1983). Tissue distribution of a coelomic-epithelium-related antigen recognized by the monoclonal antibody OC125. *Int. J. Gynecol. Pathol.,* **2,** 275.

KLUG, T.L., BAST, R.C. Jr., NILOFF, J.M., KNAPP, R.C. & ZURAWSKI, V.R. Jr. (1984). Monoclonal antibody immunoradiometric assay for an antigenic determinant (CA125) associated with human epithelial ovarian carcinomas. *Cancer Res.,* **44,** 1048.

RUIBAL, A., ENCABO, G., MARTINEZ-MIRALLES, E. & 4 others. (1984). CA125 seric levels in non-malignant pathologies. *Bull Cancer (Paris),* **71,** 145.

RUTANEN, *et al.* (1978).

RUTANEN, E.M., LINDGREN, J., SIPPONEN, P., STENMAN, U.-H., SAKSELA, E. & SEPPÄLÄ, M. (1978), Carcinoembryonic antigen in malignant and non-malignant gynecologic tumors: circulating levels and tissue localization. *Cancer,* **42,** 581.


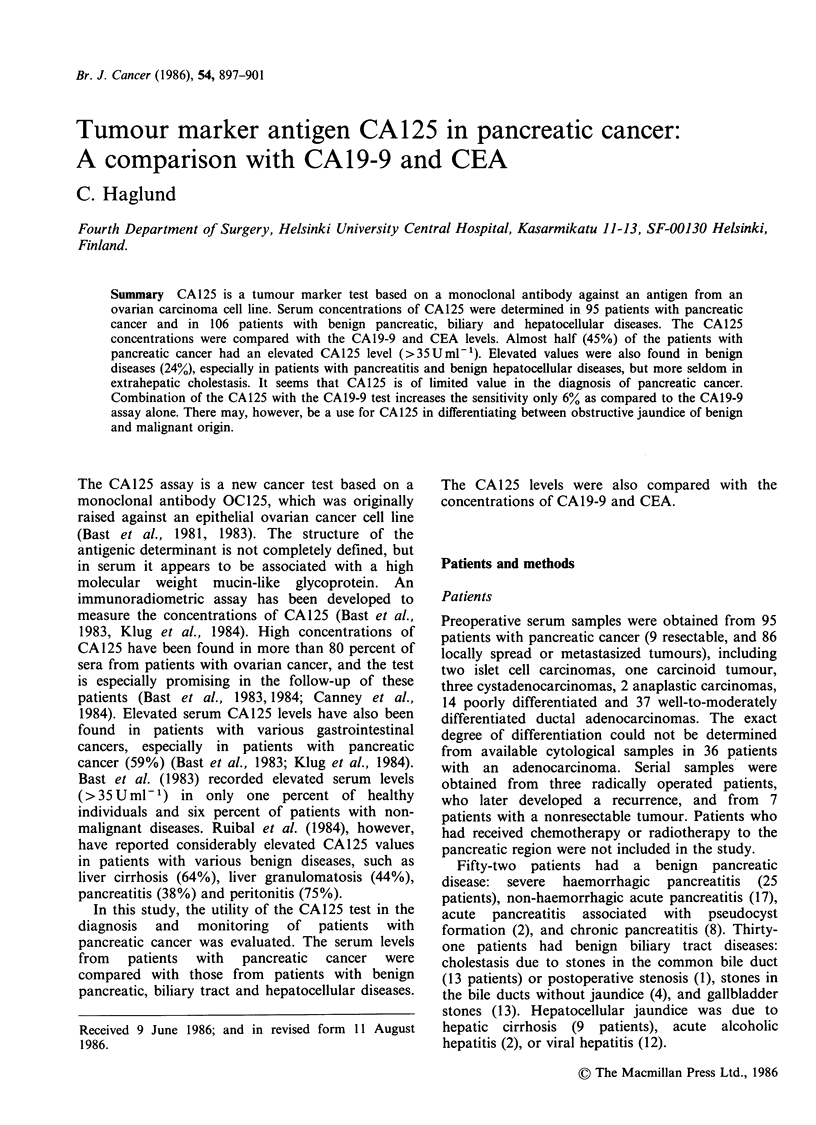

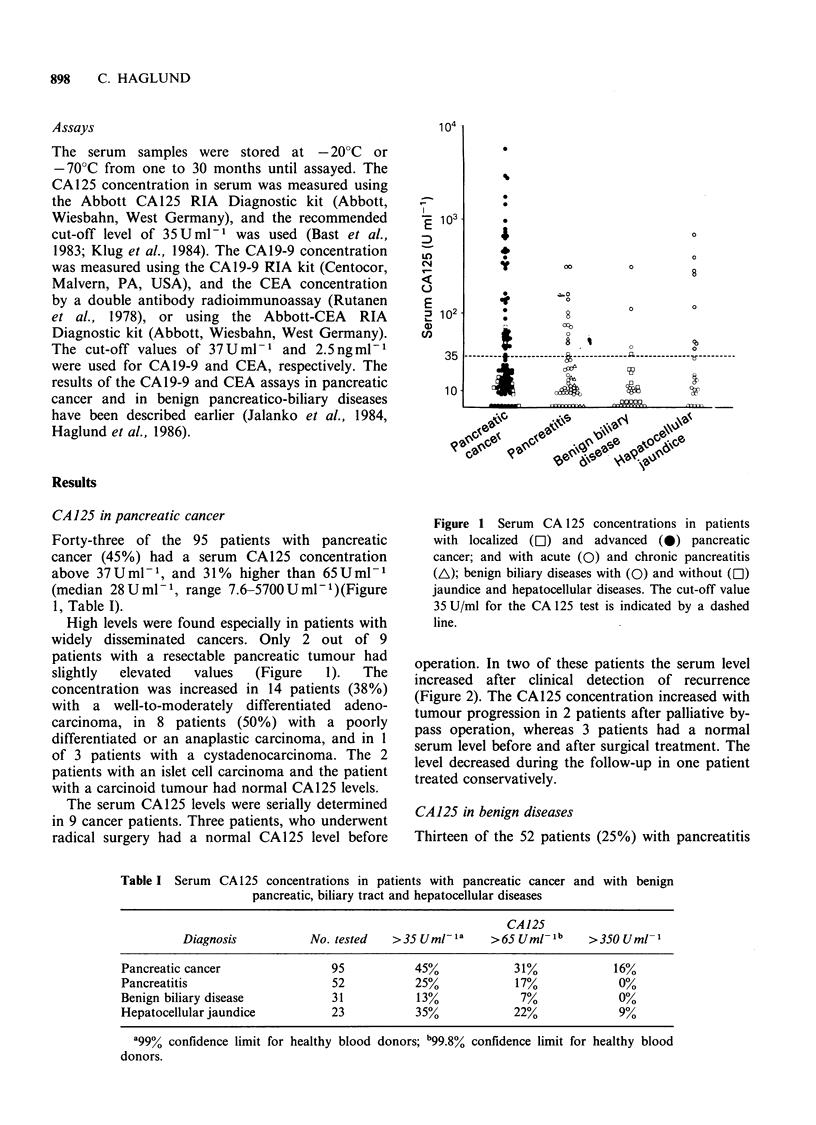

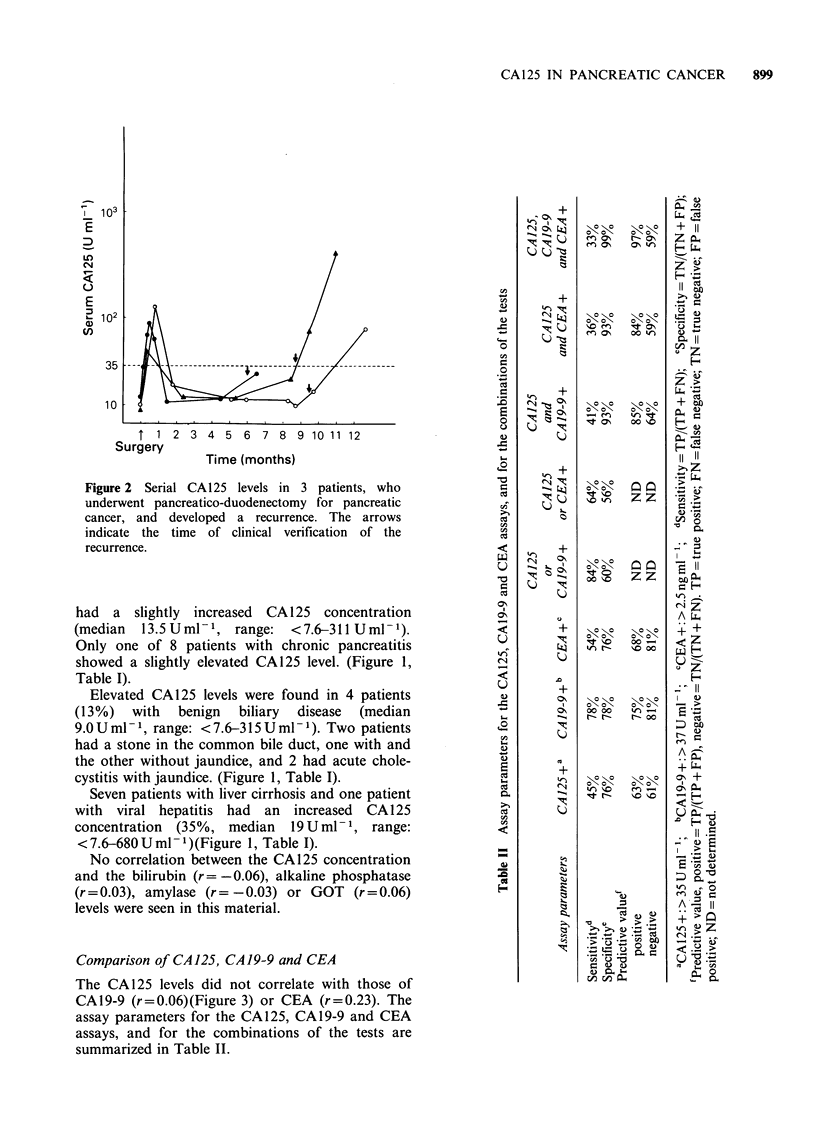

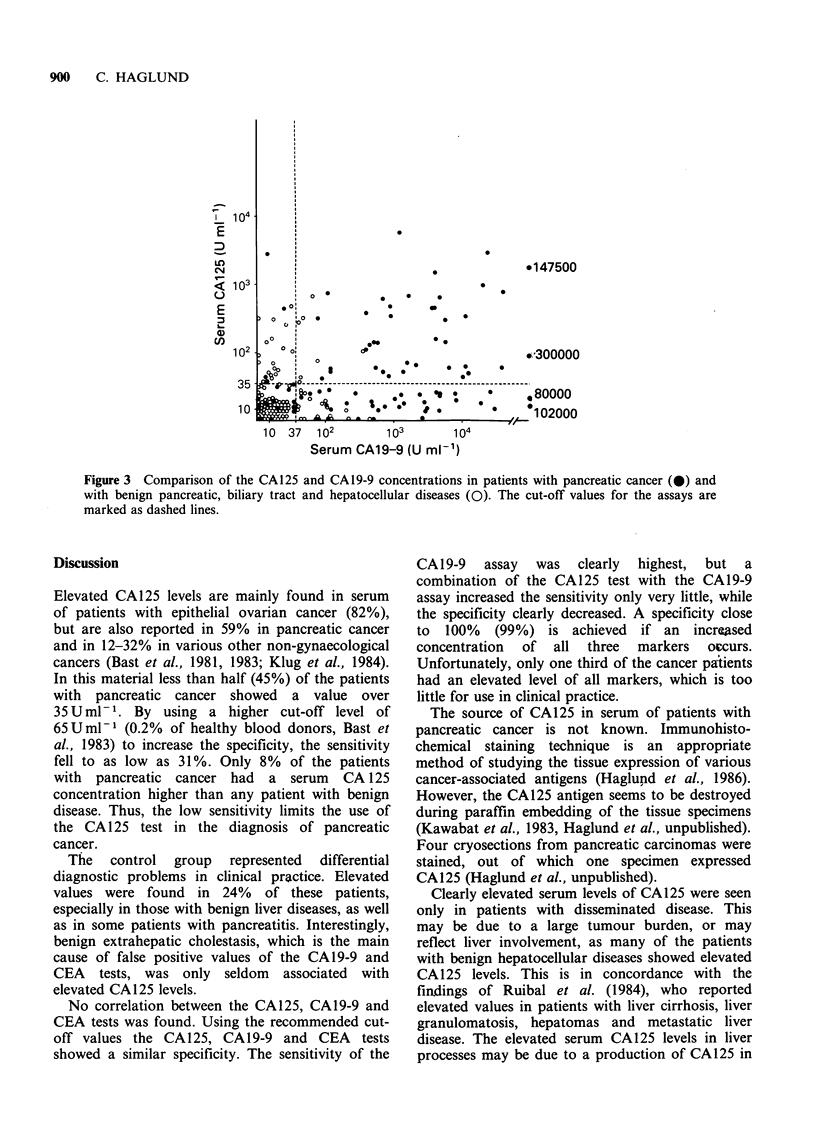

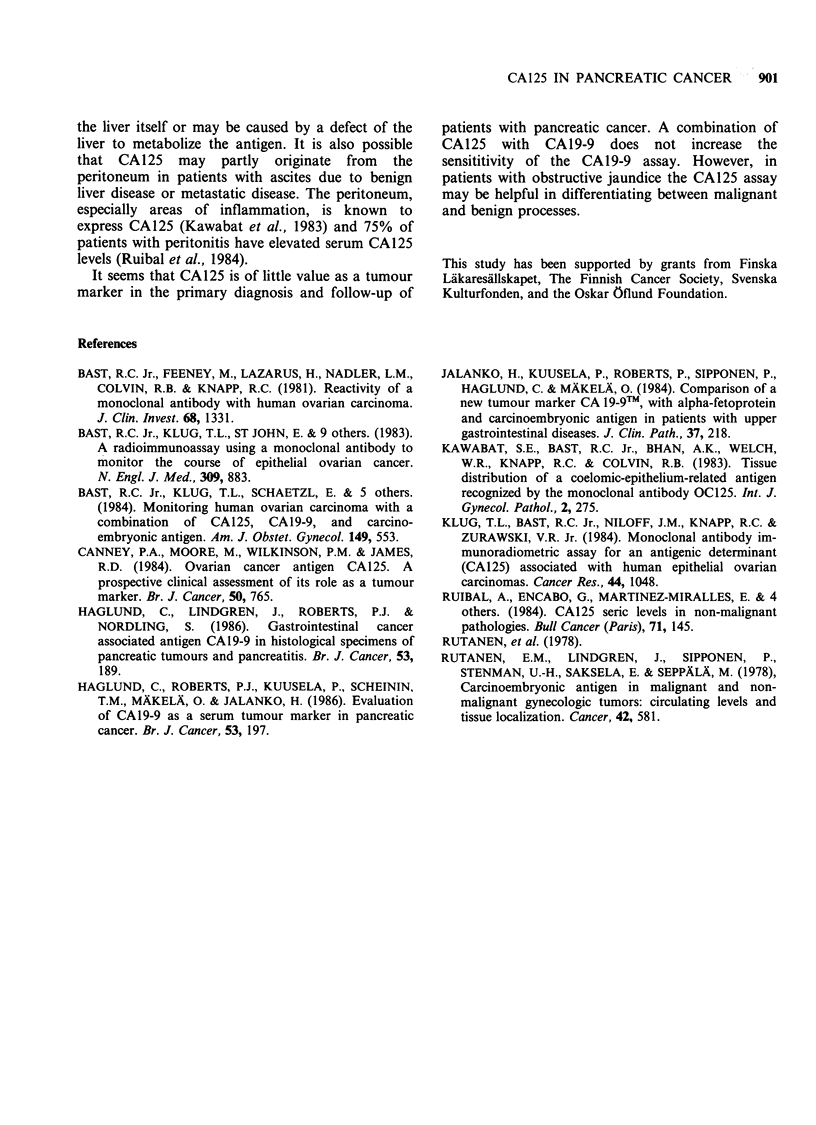


## References

[OCR_00518] Bast R. C., Feeney M., Lazarus H., Nadler L. M., Colvin R. B., Knapp R. C. (1981). Reactivity of a monoclonal antibody with human ovarian carcinoma.. J Clin Invest.

[OCR_00530] Bast R. C., Klug T. L., Schaetzl E., Lavin P., Niloff J. M., Greber T. F., Zurawski V. R., Knapp R. C. (1984). Monitoring human ovarian carcinoma with a combination of CA 125, CA 19-9, and carcinoembryonic antigen.. Am J Obstet Gynecol.

[OCR_00524] Bast R. C., Klug T. L., St John E., Jenison E., Niloff J. M., Lazarus H., Berkowitz R. S., Leavitt T., Griffiths C. T., Parker L. (1983). A radioimmunoassay using a monoclonal antibody to monitor the course of epithelial ovarian cancer.. N Engl J Med.

[OCR_00536] Canney P. A., Moore M., Wilkinson P. M., James R. D. (1984). Ovarian cancer antigen CA125: a prospective clinical assessment of its role as a tumour marker.. Br J Cancer.

[OCR_00542] Haglund C., Lindgren J., Roberts P. J., Nordling S. (1986). Gastrointestinal cancer-associated antigen CA 19-9 in histological specimens of pancreatic tumours and pancreatitis.. Br J Cancer.

[OCR_00549] Haglund C., Roberts P. J., Kuusela P., Scheinin T. M., Mäkelä O., Jalanko H. (1986). Evaluation of CA 19-9 as a serum tumour marker in pancreatic cancer.. Br J Cancer.

[OCR_00555] Jalanko H., Kuusela P., Roberts P., Sipponen P., Haglund C. A., Mäkelä O. (1984). Comparison of a new tumour marker, CA 19-9, with alpha-fetoprotein and carcinoembryonic antigen in patients with upper gastrointestinal diseases.. J Clin Pathol.

[OCR_00562] Kabawat S. E., Bast R. C., Bhan A. K., Welch W. R., Knapp R. C., Colvin R. B. (1983). Tissue distribution of a coelomic-epithelium-related antigen recognized by the monoclonal antibody OC125.. Int J Gynecol Pathol.

[OCR_00569] Klug T. L., Bast R. C., Niloff J. M., Knapp R. C., Zurawski V. R. (1984). Monoclonal antibody immunoradiometric assay for an antigenic determinant (CA 125) associated with human epithelial ovarian carcinomas.. Cancer Res.

[OCR_00576] Ruibal A., Encabo G., Martinéz-Miralles E., Murcia C., Capdevila J. A., Salgado A., Martinéz-Vasquéz J. M. (1984). CA125 seric levels in non malignant pathologies.. Bull Cancer.

[OCR_00580] Rutanen E. M., Lindgren J., Sipponen P., Stenman U. P., Saksela E., Seppäla M. (1978). Carcinoembryonic antigen in malignant and nonmalignant gynecologic tumors: circulating levels and tissue localization.. Cancer.

